# Molecular Identification and Probiotic Potential Characterization of Lactic Acid Bacteria Isolated from Human Vaginal Microbiota

**DOI:** 10.15171/apb.2018.077

**Published:** 2018-11-29

**Authors:** Yousef Nami, Babak Haghshenas, Ahmad Yari Khosroushahi

**Affiliations:** ^1^Department of Food Biotechnology, Branch for Northwest & West Region, Agricultural Biotechnology Research Institute of Iran, Agricultural Research, Education and Extension Organization (AREEO), Tabriz, Iran.; ^2^Regenerative Medicine Research Center (RMRC), Kermanshah University of Medical Sciences, Kermanshah, Iran.; ^3^Drug Applied Research Center, Tabriz University of Medical Sciences, Tabriz, Iran.; ^4^Department of Pharmacognosy, Faculty of Pharmacy, Tabriz University of Medical Sciences, Tabriz, Iran.

**Keywords:** Molecular identification, Probiotic characterization, (GTG)5 fingerprinting, Lactic acid bacteria

## Abstract

***Purpose:*** The increased demand for probiotics because of their health purposes provides the context for this study, which involves the molecular identification of lactic acid bacteria (LAB) obtained from the vaginal microbiota of healthy fertile women. The isolates were subjected for examination to prove their probiotic potential. In particular, the isolates were subjected to various tests, including acid/bile tolerance, antimicrobial activity, antibiotic susceptibility, Gram staining, and catalase enzyme activity assessment.

***Methods:*** Several methods were utilized for the molecular identification of the isolates, including ARDRA, (GTG)5-PCR fingerprinting, and the PCR sequencing of 16S-rDNA amplified fragments. Disc diffusion and well diffusion methods were used to assess antibiotic susceptibility and antibacterial activity of isolates. Tolerance to acid and bile was performed at pH 2.5 and 0.3% bile oxgall.

***Results:*** A total of 45 isolates of 88 separate organisms was selected. All of the isolates demonstrated an antibacterial effect on the exploited indicator microorganisms. All selected strains also maintained their viability at low-pH and high-bile salt conditions and exhibited abroad variation in their survival. Only the Enterococcus avium strain showed resistance to all 9 tested antibiotics. Based on the molecular identification and clustering, the 45 isolated bacteria were classified into three major groups of LAB: Enterococcus, Lactobacillus and Lactococcus.

***Conclusion:*** LAB are microorganisms that have a particularly important function in maintaining the health of the vaginal and gastrointestinal tract and in protecting it from infection by other pathogenic organisms. The isolates found to be a promising probiotic candidate by showed desirable characteristics. Therefore, strain DL3 can be used as natural food preservative with some more potential investigations.

## Introduction

The microbial species that protect the vaginal and gastrointestinal tract have an important function in maintaining the health of the vagina and in preventing infection. Over 50 microbial species have been discovered from the vaginal tract.^1,2^ Recently, microbiota, which promotes a woman’s health, has been the object of growing interest. The *Lactobacillus* species are normally present in the human vagina and have also received considerable attention because of their protective and probiotic properties.^3^ Vaginal lactobacilli include at least 70% of the total bacteria isolated from healthy women.^4^

The identification of LAB strains and their antibiotic resistance profile or their toxicity to human organs is significant. They are assessed for their potential probiotic strains intended for human consumption, aside from their health benefits. The most studied probiotics are the *Lactobacillus* and *Bifidobacterium*. However, other species, such as the *Enterococcus faecium* and *Enterococcus faecalis*, have also exhibited probiotic properties.^5^

Molecular-based methods are powerful tools of identifying probiotic bacteria and are widely used as a replacement for less superior traditional methods. Probiotics must exhibit resistance to acidic and bile-salt gastrointestinal conditions, susceptibility against antibiotics, and anti-pathogenic properties to be considered effective.

LABs are generally recognized as beneficial microorganisms. The increasing demand for them for health purposes provides the context for this study, which involves the molecular identification and in vitro characterization of LAB obtained from the vaginal microbiota of healthy fertile Iranian women. Based on our knowledge, no similar work has isolated and characterized LAB obtained from Iranian women. In this regard, this study anticipates the identification and isolation of new strains with high probiotic capability. As such, this study aimed to isolate, identify, and characterize putative probiotic bacteria from the vaginal microbiota of healthy fertile Iranian women and to analyze their probiotic potential and antimicrobial activity.

## Materials and Methods

### Microorganisms, media, and growth conditions

Vaginal swabbed specimens were taken from 40 healthy fertile Iranian women volunteers aged between 17 and 36. These specimens were isolated as sources. The LAB was isolated by spreading them on a de Man Rogosa (MRS) agar plate. They were incubated at 37 °C for 24 h in anaerobic conditions containing 5% CO_2_. Many single colonies were randomly selected and incubated in 5 mL MRS broth for 24 h. The single colonies were subjected to morphological evaluation, including gram staining, catalase test, and cell morphology. The gram-positive, catalase-negative, rod-shaped (Bacilli) and spherical (Cocci) isolates were sub-cultured and stored in an MRS broth with 25% glycerol for further experiments.

As part of our laboratory collection, the isolates were identified via phenotypic and molecular methods, including ARDRA and GTG-PCR. Furthermore, the 16S-rDNA of the LAB was amplified following their specific primers, and the amplified fragments were subsequently subjected to sequencing analysis. The stock cultures were stored at -80°C in MRS broth (MRS agar, Merck, Germany), supplemented with 25% v/v glycerol. The stock cultures of the isolated bacteria were thawed and resuspended well. About 100 µL of bacteria cultures were then inoculated into 10 mL of the MRS broth. Following a previously described method by Moghadam *et al*. (2010), the cells were cultivated at 37°C and incubated for 24 h. After incubation, the bacterial cell cultures were streaked on MRS agar and incubated for 24 h in anaerobic conditions. Single colonies were selected through an inoculating loop and incubated in MRS broth for 24 h to obtain ready-to-use-cultures.^6^

### Tolerance to low pH

Resistance to pH 3.0 is often used in *in-vitro* assays to determine resistance to low pH. Given that food stays in the gastric system for 3 h, this time limit is used in *in-vitro* acid resistance assessments.^7^ To determine the acid permanency of the isolated LAB, the method described by Claire *et al*. was applied with slight modifications.^8^ The pH of the MRS broth was adjusted to 3.0 with 1 M HCl before autoclaving, and pH 7.2 was used as the control condition. The survival of the bacterial cells was evaluated using log phase cultures (8 log_10_CFU mL^-1^) by plating them on MRS agar after 0, 1, 2, and 3 h of incubation in acidic MRS broth (pH 3.0) at 37 °C via the pour plate technique. For this purpose, the selected cells were harvested from the cultures incubated overnight through centrifugation for 10 min at 6000 ×g and 4°C. The cell pellets were washed once with phosphate buffer saline (PBS at pH 7.2). The cell pellets were then resuspended in PBS (pH 3.0) and incubated at 37°C for 3 h. Proper dilutions were performed, and the plates were incubated at 37°C in anaerobic conditions for 24 h.

### Resistance to bile salt

Tolerance to bile salts was investigated based in a formerly described method by Pereira *et al*. with slight modifications.^9^ In the experiment, 0.3% concentration of bile was applied for 4 h because the mean intestinal bile concentration is supposedly 0.3% (w/v) and the digestion time of food in the small intestine is 4 h. The MRS broth was supplemented with 0.3% (w/v) oxgall. Another MRS broth without supplement was used as the control sample and was inoculated with actively growing bacteria. The samples were incubated for 4 h at 37 °C, and bacteria maintenance was evaluated using log phase cultures (8 log_10_CFU mL^-1^) by plate count on MRS agar at time points of 0, 1, 3, and 4 h of incubation in MRS broth containing bile salts at 37 °C. During their incubation for 4 h, the viable colonies were counted every hour via the pour plate technique.

### Antimicrobial activity

The pathogenic microorganisms evaluated for the detection of antagonistic substances are mostly GIT and vaginal pathogenic microorganisms such as: *Salmonella typhimurium*, *E. coli* 026, *E. coli* 0157, *Staphylococcus aureus*, *Bacillus cereus*, *Listeria monocytogenes*, *Lactococcus Lactis* subsp. *Lactis*, *Klebsiella pneumoniae*, *Shigella flexneri*, *Pseudomonas aeroginosa*, *Candida albicans*, *Serratia marcesens*, *Enterococcus faecalis*, *Staphylococcus saprophyticus* subsp*. Saprophyticus*, and *Streptococcus mutans*. The well diffusion technique was used to detect the production of inhibitory substances in the supernatant fluids of the isolates. For the agar well diffusion assay, an overnight culture of the indicator strains was used to inoculate the appropriate agar growth media at 37 °C. Wells with a diameter of 5 mm were cut into agar plates, and 50 μl of filtered cell-free supernatant fluid obtained from the third subculture of the microorganisms grown in the MRS broth was added to each well. The supernatant fluid was obtained by growing the inhibitory producer strains overnight in the MRS broth at 37 °C. The cells were then removed via centrifugation, and the filtered supernatant fluid (using a 0.2-µm filter) was placed in the wells and was allowed to diffuse into the agar for 2 h at room temperature. Consequently, the plates were incubated at the optimum growth temperature of the indicator strains and were examined after 24 h for their inhibition zone. At the end of the incubation, the inhibition zone diameters (surrounding the wells) were measured.^10,11^

### Antibiotic susceptibility

The pattern of resistance/sensitivity to antibiotic of the LAB isolates was tested via the agar disc diffusion method.^10,12^ Antibiotic discs were employed to determine the pattern of the antibiotic resistance of the LAB strains. The discs included 10 µg of gentamycin (GE), 30 μg of chloramphenicol (C), 2 μg of clindamycin (CC), 15μg of erythromycin (ER), sulfamethoxazol (SXT), 30 μg of vancomycin (V), 30 µg of tetracycline (TE), 10 µg of penicillin (P), and 10 µg of ampicillin (AM). The LAB strains were incubated in anaerobic conditions at 37 °C and 5% CO_2_ overnight. A total of 100 μL of the diluted cultures (approximately 10^6^ to 10^7^ viable cells) was diffused in a Mueller-Hinton agar medium and the antibiotic discs were applied on the surface by using an antibiotic disc dispenser. The plates were incubated at 37 °C in anaerobic conditions and assessed after 24 h of inoculation. The inhibition zones around the discs were calculated using a digital caliper. The results were expressed in terms of resistance, moderate susceptibility, or susceptibility and were compared with the interpretative zone diameters given by the performance standards for antimicrobial disk susceptibility tests.^13^

### Genomic DNA isolation

The total DNA of the isolates from the cultures inoculated with a single colony was extracted through the procedure described by Cardinal *et al*. previously.^14^ For this purpose, a single colony was re-cultured in MRS broth for 24 h at 37 °C, and 1.5 mL of the bacterial culture was then centrifuged at 10000 ×g for 5 min. The supernatant was discarded, and the pellet was used to isolate the DNA. The extracted DNA was then suspended in 50 µL of distilled water, and all the extracted genomic DNAs of the samples were checked and visualized via 0.8% agarose gel electrophoresis. Subsequently, the gel monitoring apparatus (Biometra, Gottingen, Germany) and spectrophotometric method were used to evaluate the quality and quantity of the extracted DNA, respectively.

### Amplification of 16S-rDNA region by polymerase chain reaction

The DNA samples from the LAB isolates were subjected to PCR analysis. The amplification was conducted in a thermal cycler PTC 200 (MJC research, Waltham, USA) by using a pair of LAB-specific universal primers that have been described by Lane *et al*. (1973).^15^ PCR amplification was performed using the following temperature profile: an initial denaturation at 94 °C for 4 min, followed by 32 cycles of denaturation at 94 °C for 1 min, annealing at 58 °C for 1 min, extension at 72 °C for 1 min, and a final extension step at 72 °C for 5 min.^16^ The PCR products were resolved via electrophoresis in a 1% (w/v) agarose gel and visualized via ethidium bromide staining.

### Electrophoresis of the amplified 16S-rDNA region

The 1% solidified agarose gel (Sinagen, Iran) containing 2 µL of ethidium bromide (5 µg mL^-1^) was placed in the electrophoresis tank, and 0.5 L of 1× TAE of buffer (pH 8.0) was poured in the tank. Aliquots (10 to 12 µL) of the amplified products were mixed with 2 µL of gel-loading dye and were subjected to electrophoresis wells. A DNA size-marker (1kb, Gene Ruler DNA Ladder Plus; MBI Fermentas) was used as a molecular mass marker to ensure that the correct regions were amplified. The amplified products were electrophoresed at a constant voltage of 70 V for 1h. The amplification products were visualized in a gel documentation system (Biometra, Gottingen, Germany). The DNA fragments with a size of 1500 bp indicated the correct amplification.

### 16S-rDNA-ARDRA

Full-length 16S-rDNA gene sequences (1500 bp) were amplified with the universal genera specific Hal primers (F: 5’-AGAGTTTGATCMTGGCTCAG-3’ and R: 5’-TACCTTGTTAGGACTTCACC-3’). The PCR products were separated via electrophoresis on a 1.0% (w/v) agarose gel in 1× TAE buffer. The DNA bonds were stained with ethidium bromide. The restriction reaction with the endonuclease *Pst* I (Fermentas, St Leon-Rot, Germany) was conducted in a volume of 25 µL containing 1× incubation buffer, 0.2 U µL^-1^ of restriction enzyme, and 5 µL of the PCR products. Incubation was performed at 37°C for 2 h, and the restriction fragment patterns were detected via 2% (w/v) agarose gel electrophoresis in 1× TAE buffer. The DNA bands were quantified using 1 kb of DNA ladder (MBI Fermentas) as the molecular marker. Accordingly, all the previously reported 16S rDNA sequences in the Gene Bank were subjected to virtual digestion with *Pst* I by using the Ape software. The virtually digested pattern was compared with the experimental ARDRA result to track any difference in the digestion pattern of the unknown isolates or to identify any new isolate.

### (GTG)_5_-PCR fingerprinting

After partial clustering with ARDRA for further discrimination, (GTG)_5_ primer (5´-GTG GTG GTGGTG GTG-3´) was used as a REP-PCR oligonucleotide primer to determine the isolates in the strain level. PCR amplifications were performed using a DNA thermal cycler with a reaction volume of 25 µL, 2.5 U of Taq DNA polymerase (MP Biomedicals), 200 µM of each deoxynucleoside triphosphate, 100 ng of DNA templates, and 2µM of each primer. An initial denaturation at 94 °C for 5 min was followed by 35 cycles of denaturation for 1 min at 94 °C, annealing for 1 min at 50 °C, elongation for 3 min at 72 °C, and a final extension for 10 min at 72 °C.

### Electrophoresis and detection of GTG-PCR products 

The amplified fragments were separated on a 1.5% (w/v) agarose gel (15 × 20 cm) for 4 h at a constant voltage of 55 V in 1× TAE buffer (pH 8.0). The DNA bands were stained by ethidium bromide under UV light by using a BioDoc analyzing system (Biometra, Gottingen, Germany). The reproducibility of the (GTG)_5_-PCR protocol was investigated using four independent DNA extracts from four randomly selecting strains as templates. For the molecular marker, 1 kb of DNA molecular mass marker (Gene Ruler DNA Ladder Plus; MBI Fermentas) was used.^17^ The (GTG)_5_-PCR products were visualized after staining them with ethidium bromide under ultraviolet light, after which the digital image was captured using a CCD camera.

### 16S-rDNA gene sequencing

The PCR products from the 16S-rDNA gene (1500 bp) were amplified using the Hal primer set. The PCR products were sequenced at Sinaclone Corporation, Tehran, Iran. The sequences were then analyzed using the BLAST program of the National Center for Biotechnology Information (http://www.ncbi.nlm.nih.gov/BLAST).^18^

### Statistical analysis

GTG patterns were visualized using a gel documentation system. The images were then analyzed using a numerical taxonomy analysis program package (NTsys, Exeter software) after modification and scoring. The similarities between the strains were automatically determined by specifying the formula of Jaccard. Strain clustering was performed through the un-weighed pair group method with arithmetic averages (UPGMA). The dendrogram for the (GTG)_5_-PCR fingerprints was generated using the Pearson’s correlation similarity coefficient and the UPGMA clustering method with 1% optimization.

## Results and Discussion

### Bacteria Culture

A total of 45 isolate of 88 separate organisms, including gram-positive and catalase-negative bacteria, was selected. No gram-negative bacteria were detected in the MRS agar. Moreover, substantial catalase-positive bacteria and yeast were observed, which was probably due to vaginal contaminations. All 45 isolates, either rod- and coccus-shaped bacteria, were selected for further analysis.

The vaginal *Lactobacillus* strains of healthy women of child-bearing age is supposed to be dominated by *L. acidophilus*, followed by *L. casei* and other species.^19,20^ Our results are in agreement with those in literature. In addition to these species, others, such as *L. gasseri*, *L. fermentum*, *L. cellobiosus, L. brevis*, *L. reuteri*, *L. delbrueckii*, *L. salivarius*,* L. plantarum*, and *L. vaginalis* are reportedly the most plentiful.^21-25^ The varieties of LAB found in healthy women have been revealed to be region-specific. For instance, *L. plantarum* was reported in South Africa (25), *L. cellobiosus* in Namibia,^22^ but not in Western women, whereas *L. acidophilus* was shown to be dominated in Iranian women.

The results of this study reveal that the LAB constituting the vaginal microbiota of Iranian women would be seemed to be different from those of Korean and Western women in terms of genera and prevalence. This phenomenon may be the result of particular characteristics of vaginal epithelial cells or LABs in fermented foods, a likely origin of human LAB. For instance, the LABs in fermented foods in Western countries, such as sausages or yoghurt, are different from those found in Iranian dairy products. The link between vaginal microbiota and diet has been confirmed in a previous study.^13^ Such studies reveal that orally administered probiotics may appear in the vaginal fluid. As such, we suggest that one of the sources of vaginal microbiota is the LAB present in fermented foods.

### Resistance to low pH

The survival rates of the 45 different LAB strains at acidic conditions (pH 3.0) are illustrated in Table 1. In general, the viable count (log CFU mL^-1^) was substantially decreased especially after 3 h. *Lactobacillus plantarum* 5BL and *Lactococcus lactis* 2HL showed the highest viability followed by *Lactococcus lactis* 17YLAC, *Lactobacillus casei* 17Y, *Enterococcus faecium* 36Y, *E. hirae* 20HL, and *E. pseudoavium* 5HL. *E. faecalis* 19HS exhibited the lowest viability after 3 h. Based on the results, all 45 selected strains retained their viability even after 3 h of exposure to pH 3.0.Notably, a broad variation in survival was observed at this condition. Most of the strains showed a high survival rate (more than 50%) at pH 3.0 after 1and 2h. After 3 h, only 30 out of the 45 strains showed a survival rate of more than 50%. The minimum survival rate after 3 h was related to the following isolates: *E. faecalis* 19HS, *E. faecalis* 1H, *E. avium* 19Y, and *E. malodoratus* 10HS, reaching up to 18%, 21%, 22%, and 24% viability, respectively. The maximum viability of the enterococci was 78%. As such, the viability of the enterococci strains was between 18 and 78% after 3 h. The diverse survival rates of the *Enterococcus faecalis* strains (from 18 to 73% viability) suggest that their survival ability is a strain-specific property.

Furthermore, the viability of the *Lactococcus* strains was between 62 and 88% after 3 h. The results indicate that the viability of lactococci is more than that of the enterococci in pH 3 after 3 h. By contrast, the results also show that lactococci are more resistant than enterococci to low pH.

Furthermore, the survival rate of lactobacilli was between 53 and 88% after 3 h, which means that lactobacilli are also more resistant than enterococci to low pH. The maximum viability after 3 h was 88%, a rate obtained by two strains, namely, the *Lactobacillus plantarum* and *Lactococcus lactis*. Consequently, the 13 strains that were most resistant to low pH and bile oxgall were selected for further analysis. All 13 strains are very stable in pH 3.0, which means that these isolates can survive in this pH value.

### Tolerance to bile salt

The viable count of 45 different LAB strains including enterococci, lactococci, and lactobacilli at bile concentration of 0.3% is shown in Table 2. All 45LAB strains demonstrated different activities at 0.3% bile concentration and 4 h of incubation. The results showed the reasonable growth during the incubation period for a majority of the selected strains. *Lactococcus lactis* 2HL showed the highest growth followed by *Lactobacillus plantarum* 5BL and *Enterococcus avium* 7BL at 0.3% bile concentration after 4 h of incubation. By contrast, *Enterococcus faecalis* 1H showed the lowest viability followed by *E. avium* 19Y and *E. faecalis* 19HS, after 4 h of incubation.

**Table 1:** Survival rate of lactic acid bacteria strains after incubation at pH value 3.0

**Strain**	**Low pH (SR%)** ^a^ **After 3 h**	**Final counts (log cfu mL^-1^) after incubation at:**			
		**0 h**	**1 h**	**2 h**	**3 h**
20BL (*Enterococcus faecium*) 15BL (*Enterococcus faecium*) 36Y (*Enterococcus faecium*) 15BS (*Enterococcus faecium*) 20BS (*Enterococcus malodoratus*) 19B (*Enterococcus malodoratus*) 14H (*Enterococcus malodoratus*) 10HS (*Enterococcus malodoratus*) 6BL (*Enterococcus malodoratus*) 13B (*Enterococcus faecalis*) 1H (*Enterococcus faecalis*) 9B (*Enterococcus faecalis*) 17H (*Enterococcus faecalis*) 19HS (*Enterococcus faecalis*) 16H (*Enterococcus faecalis*) 17BL (*Enterococcus faecalis*) 1HL (*Enterococcus faecalis*) 12HL (*Enterococcus durans*) 6HL (*Enterococcus durans*) 10HL (*Enterococcus durans*) 7BL (*Enterococcus avium*) 19YC (*Enterococcus avium*) 18HL (*Enterococcus avium*) 19Y (*Enterococcus avium*) 7BS (*Lactobacillus acidophilus*) 15HL (*Lactobacillus acidophilus*) 36YL (*Lactobacillus acidophilus*) 8BS (*Enterococcus gilvus*) 15HS (*Enterococcus gilvus*) 20HL (*Enterococcus hirae*) 2BS (*Enterococcus hirae*) 14BS (*Enterococcus hirae*) 20HS (*Enterococcus hirae*) 5HL (*Enterococcus pseudoavium*) 12HS (*Enterococcus pseudoavium*) 5BL (*Lactobacillus plantarum*) 18HS (*Lactobacillus casei*) 17Y (*Lactobacillus casei*) 1BL (*Enterococcus lactis*) 7HL (*Enterococcus lactis*) 16B (*Enterococcus lactis*) 2BL (*Enterococcus lactis*) 2HL (*Lactococcus lactis*) 17YLAC (*Lactococcus lactis*) 13HL (*Lactococcus lactis*)	48 56 78 53 58 41 37 24 47 52 21 49 61 18 73 51 33 54 63 58 71 46 59 22 64 72 81 74 62 77 41 61 30 75 59 88 79 53 33 47 58 67 88 84 62	9.02 9.01 9.03 8.84 8.96 9.01 9.05 8.12 7.36 8.97 9.06 8.58 8.81 7.03 8.93 8.85 9.06 7.92 9.29 8.03 7.90 8.04 7.98 7.77 8.16 7.57 9.91 9.41 8.27 8.92 9.39 7.85 7.99 8.36 7.77 9.70 9.48 8.72 8.90 9.04 9.58 7.28 7.98 9.05 9.47	5.92 6.37 7.95 5.93 6.71 5.12 4.76 3.01 4.94 5.82 3.09 5.46 7.04 2.49 7.58 5.86 6.94 5.50 6.91 5.99 6.48 4.95 5.97 2.98 6.58 6.78 8.83 7.99 6.85 7.83 4.94 5.80 3.91 6.94 5.33 8.90 8.02 5.69 4.27 5.83 5.69 6.25 7.81 8.68 7.63	5.01 5.59 7.41 5.28 5.89 4.36 4.01 2.55 4.11 5.13 2.34 4.97 6.22 1.94 7.03 5.07 6.58 4.99 6.23 5.20 5.92 4.03 5.22 2.37 6.02 6.13 8.45 7.34 6.11 7.04 4.01 5.13 3.08 6.59 4.96 8.63 7.78 5.12 3.42 4.91 4.99 5.82 7.37 8.02 6.74	4.33 5.04 7.04 4.68 5.19 3.69 3.34 1.94 3.45 4.66 1.90 4.20 5.37 1.26 6.51 4.51 5.97 4.27 5.85 4.65 5.60 3.69 4.70 1.70 5.22 5.45 8.02 6.96 5.12 6.86 3.65 4.78 2.39 6.27 4.58 8.52 7.48 4.62 2.93 4.24 5.55 4.88 7.02 7.60 5.87

^a^ Survival rate after 3 hour in pH value 3.0

**Table 2:** Survival rate of lactic acid bacteria strains after incubation at 0.3 % bile salt (Oxgall)

**Strain**	**Bile salts (SR%)** ^a^ **after 4 h**	**Final counts (log cfu mL^-1^) after incubation at:**				
		**0 h**	**1 h**	**2 h**	**3h**	**4 h**
20BL (*Enterococcus faecium*) 15BL (*Enterococcus faecium*) 36Y (*Enterococcus faecium*) 15BS (*Enterococcus faecium*) 20BS (*Enterococcus malodoratus*) 19B (*Enterococcus malodoratus*) 14H (*Enterococcus malodoratus*) 10HS (*Enterococcus malodoratus*) 6BL (*Enterococcus malodoratus*) 13B (*Enterococcus faecalis*) 1H (*Enterococcus faecalis*) 9B (*Enterococcus faecalis*) 17H (*Enterococcus faecalis*) 19HS (*Enterococcus faecalis*) 16H (*Enterococcus faecalis*) 17BL (*Enterococcus faecalis*) 1HL (*Enterococcus faecalis*) 12HL (*Enterococcus durans*) 6HL (*Enterococcus durans*) 10HL (*Enterococcus durans*) 7BL (*Enterococcus avium*) 19YC (*Enterococcus avium*) 18HL (*Enterococcus avium*) 19Y (*Enterococcus avium*) 7BS (*Lactobacillus acidophilus*) 15HL (*Lactobacillus acidophilus*) 36YL (*Lactobacillus acidophilus*) 8BS (*Enterococcus gilvus*) 15HS (*Enterococcus gilvus*) 20HL (*Enterococcus hirae*) 2BS (*Enterococcus hirae*) 14BS (*Enterococcus hirae*) 20HS (*Enterococcus hirae*) 5HL (*Enterococcus pseudoavium*) 12HS (*Enterococcus pseudoavium*) 5BL (*Lactobacillus plantarum*) 17Y (*Lactobacillus casei*) 18HS (*Lactobacillus casei*) 1BL (*Enterococcus lactis*) 7HL (*Enterococcus lactis*) 16B (*Enterococcus lactis*) 2BL (*Enterococcus lactis*) 2HL (*Lactococcus lactis*) 17YLAC (*Lactococcus lactis*) 13HL (*Lactococcus lactis*)	68 76 88 73 78 61 57 44 57 72 31 61 74 34 89 70 53 74 86 68 91 66 79 32 72 81 89 87 73 90 57 78 43 87 67 91 88 72 42 59 68 89 93 88 76	9.12 8.02 8.34 8.21 7.97 8.93 8.06 9.12 8.32 7.64 8.56 7.94 7.98 8.58 9.05 8.45 8.27 8.86 9.15 8.18 7.78 9.22 9.09 7.93 8.13 7.35 8.38 9.27 7.25 9.23 8.74 7.83 7.82 8.83 7.96 9.80 9.11 8.88 7.90 8.08 8.32 7.56 7.48 8.95 8.33	7.73 7.47 8.05 6.99 7.01 6.87 5.96 6.11 5.72 6.31 3.79 5.92 7.17 4.56 8.61 7.26 5.66 7.44 8.41 6.69 7.42 6.63 7.94 3.98 6.72 6.77 7.81 8.37 6.95 8.94 5.87 6.38 4.46 8.15 6.31 9.50 8.42 7.44 4.95 6.08 6.57 7.07 7.12 8.16 6.97	7.21 7.03 7.73 6.53 6.73 6.21 5.55 5.55 5.47 6.04 3.36 5.57 6.89 3.92 8.49 6.82 5.14 7.19 8.26 6.34 7.29 6.48 7.68 3.58 6.37 6.42 7.68 8.29 6.89 8.74 5.56 6.27 4.09 8.02 5.96 9.30 8.29 6.97 4.43 5.66 6.32 6.99 7.05 8.09 6.83	6.84 6.59 7.57 6.18 6.48 5.82 5.01 4.85 5.15 5.86 2.94 5.20 6.33 3.55 8.34 6.47 4.88 6.97 8.12 5.99 7.16 6.31 7.41 3.03 6.09 6.14 7.54 8.14 6.84 8.57 5.22 6.19 3.71 7.90 5.72 9.17 8.18 6.63 3.87 5.12 5.91 6.84 6.99 7.96 6.54	6.20 6.09 7.34 5.99 6.22 5.45 4.59 4.01 4.74 5.50 2.65 4.84 5.91 2.92 8.05 5.92 4.38 6.56 7.87 5.56 7.08 6.09 7.18 2.54 5.85 5.95 7.46 8.06 6.77 8.31 4.98 6.11 3.36 7.68 5.33 9.02 8.02 6.39 3.32 4.77 5.66 6.73 6.96 7.88 6.33

^a^ Survival rate after 4 hour in 0.3% bile concentration Experiments were performed in triplicate.

Furthermore, the survival rate of the enterococci ranged from 32 to 90%, whereas the viability of the lactococci and lactobacilli ranged 76 to 93% and 72 to 91%, respectively. Based on these results, the tolerance of the lactococci and lactobacilli to bile is more than that of the enterococci. *Lactobacillus plantarum* 5BL, *Lactococcus lactis* 2HL, *Enterococcus lactis* 2BL, *E. faecium* 36Y, *E. hirae* 20HL, *E. avium* 7BL, *E. pseudoavium* 5HL, *Lactobacillus casei* 18HS, *E. gilvus* 8BS, *Lactobacillus acidophilus* 36YL, *E. durans* 6HL, *E. faecalis* 16H, and *E. malodoratus* 20BS showed excellent acid and bile tolerance. Thus, in this study, all thirteen robust LAB strains were selected for their antimicrobial and antibiotic activities to confirm further their properties as probiotic bacteria and to evaluate their potential therapeutic uses.

### Antimicrobial activity

The diameter of the inhibition zones indicated that all of the isolates had an antibacterial effect on the indicator microorganisms. The tests were applied three times, and the average of the diameters of the zones was obtained. The results showed that four vaginal isolates, including *Lactobacillus plantarum* 5BL, *Lactobacillus acidophilus* 36YL, *Lactobacillus casei* 18HS, and *Lactococcus lactis* 2HL were active against *Gardnerella vaginalis*, which is the microorganism commonly associated with bacterial vaginosis (Table 3). Most of the 13 tested LAB strains did not inhibit the growth of *Pseudomonas aeroginosa* and *Candida albicans*, except strains 2HL, which belongs to *Lactococcus lactis*. This strain was also active against all tested indicator microorganisms. Moreover, the growth of the *Salmonella typhimurium* was suppressed by seven strains, namely, *Lactococcus lactis* 2HL, *Lactobacillus plantarum* 5BL, *Enterococcus durans* 6HL, *E. faecalis* 16H, *E. hirae* 20HL, *Lactobacillus acidophilus* 36Y and 36YL. The results revealed that all three genera of LAB assessed in this study (*Lactococcus*, *Enterococcus*, and *Lactobacillus*) suppressed the growth of *Salmonella typhimurium*.

Additionally, 12 out of the 13 LAB evaluated in this study had good inhibitory effects on *Staphylococcus aureus*. Only the strain 7BL had an inhibitory effect on *S. aureus*, whereas strains 2BL, 5HL, 6HL, 7BL, 36Y, 16H, 20HL, and 20BS were not able to inhibit *Staphylococcus saprophyticus* growth. However, all *Enterococcus* and *Lactococcus* strains inhibited the growth of *Listeria monocytogenes*. Two *Lactobacillus* strains, 18HS and 36YL, did not inhibit the growth of *Listeria monocytogenes*.

Another investigated indicator microorganism in this study was *Shigella flexneri*. The results show that most of the LAB inhibited *Shigella flexneri*, except 7BL and 36YL. Moreover, the inhibitory effects of the strains on *Enterococcus faecalis*, *Bacillus cereus*, and *Klebsiella pneumoniae* were the same. The growth of these three organisms was inhibited by only two out of thirteen LAB strains, 6HL and 20HL.

Barzegari and Saei suggested the isolation of probiotics with respect to native microbiome and, in the interest of efficacy, their application in a similar population. The efficiency of a probiotic can be determined in the target population before usage to guarantee its health and therapeutic benefits.^26^ In the present study, two native (*Escherichia coli* 026) and non-native (*Escherichia coli* 0157) pathogenic organisms were assessed. All tested LAB, except strain *Enterococcus avium* 7BL, inhibited the growth of *Escherichia coli* 026, whereas strains 2BL, 6HL, 8BS, 36YL, and 18BS did not exhibit any inhibitory effects on the growth of *Escherichia coli* 0157, which confirmed Barzegari and Saei’s suggestion.

**Table 3:** The inhibitory effect of selected lactic acid bacteria against pathogenic and non-pathogenic bacteria

**Indicator organisms**	**Selected lactic acid bacteria**												
	**2HL**	**2BL**	**5BL**	**5HL**	**6HL**	**7BL**	**8BS**	**36Y**	**20BS**	**36YL**	**20HL**	**16H**	**18HS**
*Salmonella typhimurium* *Escherichia coli* 026 *Escherichia. coli* 0157 *Staphylococcus aureus* *Bacillus cereus* *Gardnerella vaginalis* *Listeria monocytogenes* *Klebsiella pneumoniae* *Enterococcus faecalis* *Shigella flexneri* *Pseudomonas aeroginosa* *Candida albicans* *Serratia marcesens* *Streptococcus mutans* *Staphylococcus saprophyticus*	13 15 11 10 13 21 9 10 12 13 11 8 9 14 11	0 10 0 11 9 0 17 10 18 11 0 0 0 5 0	16 18 16 15 8 11 15 9 11 15 0 0 16 17 8	0 15 13 14 17 0 12 19 10 13 0 0 3 0 0	3 5 0 3 0 0 2 0 0 7 0 0 0 0 0	0 0 3 0 10 0 6 6 8 0 0 0 2 0 0	0 8 0 7 6 0 15 7 11 6 0 0 0 5 4	14 24 15 15 5 0 3 17 7 17 0 0 17 15 0	0 21 7 14 13 0 6 12 11 3 0 0 6 0 0	13 7 0 14 2 16 0 11 5 0 0 0 12 0 13	19 18 16 16 0 0 7 0 0 14 0 0 14 0 0	17 19 14 17 13 0 14 14 7 16 0 0 18 20 0	0 19 0 14 8 12 0 15 14 7 0 0 0 12 7

0 (Resistant); 0-4 mm (Semi Resistant); 4-8 mm (Semi Susceptible); 8-12 mm (Susceptible); >12 mm (Extra Susceptible)

### Antibiotic susceptibility

Our findings showed the resistance of strain *Enterococcus avium* 7BL to all nine tested antibiotics. The resistance of the strain is intrinsic and non-transmissible. Nevertheless, the antibiotic resistance of all LAB are not intrinsic, especially that of lactobacilli. The resistance of some LAB may possibly be plasmid-encoded. As such, their antibiotic resistance was carefully evaluated before use as commercial probiotic strains. By contrast, all tested antibiotics suppressed the growth of the strains *Enterococcus durans* 6HL and *Lactococcus lactis* 2HL, which means that these strains are sensitive to all nine tested antibiotics.

In addition, most of the *Enterococcus* strains were resistant to erythromycin, except *Enterococcus durans* 6HL, which was sensitive. *Enterococcus hirae* 20HL and *Enterococcus malodoratus* 20BS were intermediate, whereas all lactobacilli and lactococci were sensitive to erythromycin. As shown in Table 4, all *Lactobacillus* and *Lactococcus* strains were susceptible to h-Lactam antibiotics (penicillin and ampicillin) and gram-positive spectrum antibiotic (erythromycin), except strain *Lactobacillus plantarum* 5BL, which was resistant to penicillin.

Vancomycin resistance is the most important concern in terms of antibiotic resistance because vancomycin is one of the last antibiotics that is largely effective against clinical infections caused by multidrug-resistant pathogens.^27,28^ However, specific LAB, including strains of *L. rhamnosus*, *L. casei*, *L. plantarum*, and *Leuconostoc* spp. and pediococci, are resistant to vancomycin. Such resistance is commonly intrinsic, which is chromosomally encoded and is non-transmissible.^29^ In the present study, we found that *Lactobacillus casei* 18HS and the *Enterococcus* strains (*Enterococcus avium* 7BL, *Enterococcus hirae* 20HL) were resistant to vancomycin (Table 4). The results are similar to those of Tynkkynen *et al*. (1998) and Lim *et al*. (1993).^30,31^

**Table 4:** Antibiotic susceptibility profiles of test strains

**Strains Code** **(Species)**	**Antibiotics**								
	**AM** ^a^	**P**	**ER**	**V**	**C**	**TE**	**GE**	**CC**	**SLX**
*Enterococcus durans* 6HL	S	S	S	S	S	S	S	S	S
*Enterococcus hirae* 20HL	S	S	I	S	S	R	R	S	R
*Enterococcus faecalis* 16H	S	S	R	S	I	S	S	R	S
*Enterococcus pseudoavium* 5HL	S	S	R	S	S	I	R	I	R
*Enterococcus hirae* 20HL	R	S	R	R	R	S	S	S	I
*Lactobacillus acidophilus* 36YL	S	S	S	S	R	S	S	I	R
*Lactobacillus plantarum* 5BL	S	R	S	S	R	I	S	S	S
*Enterococcus gilvus* 15HS	S	S	S	S	I	S	I	R	R
*Enterococcus avium* 7BL	R	R	R	R	R	R	R	R	R
*Enterococcus lactis* 2BL	S	S	R	S	S	R	S	I	S
*Lactococcus lactis* 2HL	S	S	S	S	S	S	S	S	S
*Enterococcus malodoratus* 20BS	R	S	I	S	I	I	S	R	S
*Lactobacillus casei* 18HS	S	S	S	R	S	S	R	S	S

R, resistant; I, intermediate; S, sensitive ^a^AM, ampicillin; P, penicillin; ER, erythromycin; V, vancomycin; C, chloramphenicol; CC, clindamycin; GE, gentamycin; TE, tetracycline; SLX, sulfamethoxazol

### Identification of LABs through ARDRA analysis

**Figure 1:**
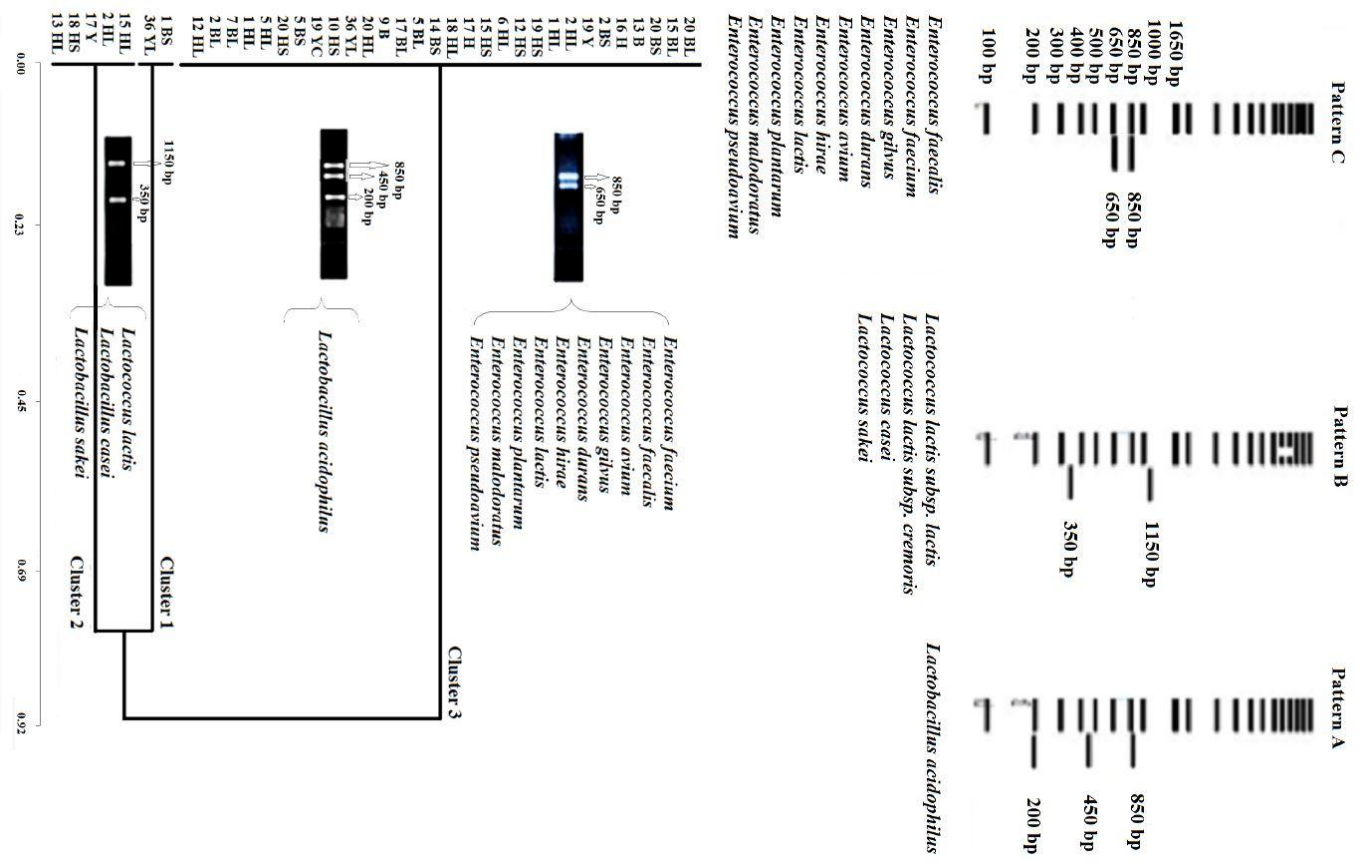
The ARDERA analysis results. (a) the virtually cleaving pattern of standard bacteria species in GenBank as a reference species by using the *Pst* I enzyme and (b) three distinct groups obtained by ARDRA clustering with higher homology to *Lactobacillus*, *Lactococcus* and *Enterococcus* strains.

In this study, new isolates were identified by comparing the isolates with the bioinformatically and virtually digested pattern from the GenBank sequence (Figure 1a). Such virtual implementation is applicable through GeneDoc. This modified technique demonstrated high discrimination efficiency with the *Pst* I enzyme.

Similarly, the same profile was observed when the 16S-rDNA amplicon of the 14 isolates were cleaved using the Pst I enzyme (Figure 1b). A comparison of the virtual ARDRA profiles with the unknown isolates revealed three distinct isolates with a higher homology to the *Lactobacillus*, *Lactococcus*, and *Enterococcus* strains. This simple and widely applicable system can be considered as an alternative virtual technique for the conventional ARDRA method because of its high correlation. Given the lack of restriction sites in the variable part of the different isolates, the ARDRA technique was able to divide the 45 isolates into only 3 main clusters namely, *Lactobacillus*, *Lactococcus*, and *Enterococcus*. The results show that the isolates classified in group one may possibly belong to species, such as *E. faecium*, *E. faecalis*, *E. avium*, *E. pseudoavium*, *E. gilvus*, *E. malodoratus*, *E. hirae*, *E. durans*, *E. lactis*, and *L. plantarum*. Moreover, the isolates in group two may possibly be *Lactobacillus acidophilus*, whereas the species in group three may be *Lactococcus lactis*, *Lactobacillus casei*, and *Lactobacillus sakei*.

Although molecular identification systems, such as ARDRA, RAPD, and ERIC-PCR, display a high effectiveness in the clustering of new strains, they endure the intricacy and complexity of interpretation and the lack of proper reference strains. By contrast, in several cases, the allocation of proper reference strains imposed by the preparation of authentic and reliable reference strains, limited the number of in-use reference bacteria and the lack of similarity between new strains and reference bacteria. To deal with these problems, new isolates were determined in this study by comparing them with the bioinformatically and virtually digested pattern from the GenBank sequence. Such virtual implementation is applicable through the use of software, such as GeneDoc. This modified technique demonstrated high discrimination efficiency with the *Pst* I enzyme.

### Identification of LAB by (GTG)_5_-PCR analysis

**Figure 2:**
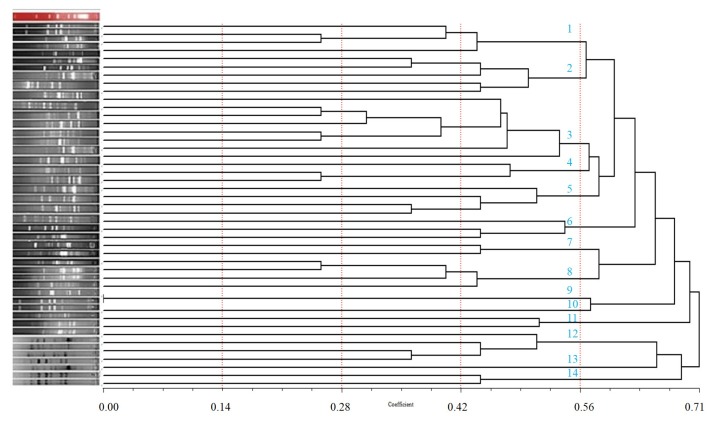
Dendrogram generated after cluster analysis of the digitized (GTG)_5_-PCR fingerprints of the vaginal lactic acid bacteria strains. The dendrogram was constructed using the un-weighted pair-group method using arithmetic averages with correlation levels expressed as percentage values of the Pearson correlation coefficient.

The GTG-primer yielded the lowest number of bands from 6 to a maximum of15 visualized PCR products with an average of 11. This primer generated DNA fragments with sizes ranging from 300 to 6000 bp. The banding patterns were standardized with a 1-kb DNA ladder and imaged via LabWorks^TM^ image acquisition. Hierarchical cluster analysis was then performed using the (GTG)_5_-PCR banding patterns (SPSS, ver. 19.0 for Windows). The similarities between the fingerprints were calculated using the Pearson’s product moment correlation coefficient. A dendrogram was constructed using the UPGMA algorithm. The dendrogram obtained from the PCR pattern shows that at a similarity level of 20%, 14 distinct clusters were observed and each cluster appeared to be the representative of different species (Figure 2).

Consequently, a group of 45 unidentified LAB isolated from the vaginas of Iranian women were included in this study to assess this technique for its capacity to identify unknown isolates. These isolated 45 strains were well-identified and characterized via (GTG)_5_-PCR fingerprinting. All strains were clearly grouped into well-separated clusters, each representing a single species. Of the total, 8strains were separated in a cluster representing *E. faecalis*; 4strains matched *E. faecium*; 5strains *E. malodoratus*; 3 strains *E. durans*, *Lactobacillus acidophilus* and *Lactococcus lactis*; 4 strains *E. avium*, *E. hirae* and *E. lactis* strains; and 2 strains *E. gilvus*, *E. pseudoavium* and *Lactobacillus casei* (Figure 2).

Thereafter, the isolated strains were reanalyzed to verify the reproducibility of the (GTG)_5_-PCR fingerprinting (data not shown). All strains provided the same band patterns without qualitative differences as a result of missing bands. However, differences in the band intensity of several fingerprints were observed.

Several different rep-PCR fingerprinting primers exist, including BOXA1R, GTG, REP1R-I, REP2-I, and ERIC. For the assessment of the rep-PCR fingerprinting method, one single oligonucleotide primer, (GTG)_5_, was initially tested for its ability to type a subset of the 45 LAB isolates obtained from the vaginas of healthy fertile Iranian women. The (GTG)_5_ primer was chosen to classify the LAB because of its ability to generate banding patterns with the highest complexity (32). Moreover, (GTG)_5_ sequences appear to be extensively distributed in the genomes of different bacterial groups.

To date, some studies on the usage of the (GTG)_5_ primer for rep-PCR fingerprinting are available.^32^ For example, Svec *et al*. used the (GTG)_5_ primer to type *Enterococcus* species.^33^ Gevers *et al.* used (GTG)_5_ to evaluate *Lactobacillus* species.^34^ De Vuyst *et al.* used (GTG)_5_ to identify and classify acetic acid bacteria.^35^ Nick *et al.* confirmed the usefulness of the (GTG)_5_ primer in typing rhizobial strains.^36^

### Identification of LABs by sequencing of 16S-rDNA

Based on the16S-rDNA identification, the 45 isolated bacteria were classified into three major groups of LAB: enterococci, lactobacilli, and lactococci. The isolates classified as LAB were also separated and identified via sequencing. After sequencing, the strains belonging to the *Enterococcus* genus were categorized to nine different species: *E. lactis*, *E. pseudoavium*, *E. hirae*, *E. gilvus*, *E. avium*, *E. durans*, *E. faecalis*, *E. malodoratus*, and *E. faecium*. Moreover, the lactobacilli were classified into three diverse species: *Lactobacillus casei*, *Lactobacillus acidophilus*, and *Lactobacillus plantarum*. The lactococci were classified only into one species: *Lactococcus lactis*, with two subspecies named as *lactis* and *cremoris*. Furthermore, the majority of these strains belongs to the *Enterococcus faecalis* and account for about 19% of the total strains.

The analysis of the 16S-rDNA pattern assists in the identification of probiotic bacteria genus according to FAO/WHO guidelines.^37^ The use of DNA sequences encoding 16S-rDNA has been proposed as a proper substitute;^38^ however, DNA-DNA hybridization remains the golden standard procedure in identifying strains. Given that this methodology is laborious and costly, it requires a large collection of reference strains. The identification of new strains of probiotic bacteria have been frequently described in literature by amplifying the16S-rDNA gene, which is highly conserved in some parts. However, it has highly variable regions that can provide a strain-specific signature. Therefore, to confirm the presence of *Lactobacillus*, *Lactococcus*, and *Enterococcus* in the genus level, the PCR amplification of 16S-rDNA gene was performed, and the PCR products were sequenced.

As previously estimated, 80% of the mechanism of the human immune system is related to live microbiota, and 20% is dependent on the intrinsic immune system of the body. Moreover, the microbiome of each community is formed as a result of many years of mutualism between the individuals and their native microbiota. Therefore, the investigation of the microbiome of each community is important to gather information specific to each community across the world.^39^ Considering this trend of research in the field of microbiology, an immense quantity of data is necessary to establish perfect patterns that will reveal the relationship between changes in microbiomes and health/disease symptoms. The data obtained from this kind of investigation, combined with the outcomes of the metagenome project, can help assess the design of suitable microbiome fingerprinting patterns in the future. These patterns may show the relationship between disease occurrence and microbiome disorders. Thus, we may be able to predict the occurrence of diseases by examining the disorder in microbiome maps in the near future.

## Acknowledgments

The financial support of Agricultural Biotechnology Research Institute of Iran (ABRII) is gratefully acknowledged.

## Ethical Issues

No ethical issues to be promulgated.

## Conflict of Interest

The authors declare that there are no conﬂicts of interests. No writing assistance was utilized in the production of this manuscript.
